# The Germ Theory of Typhoid Fever and Treatment of This Disease by Salicylite of Bismuth

**Published:** 1883-09

**Authors:** 


### THE GERM THEORY OF TYPHOID FEVER, AND THE TREATMENT OF THIS DISEASE BY SALICYLATE OF BISMUTH.

The tendency of the age is undoubtedly to account for all the contagious febrile affections, as well as many chronic diseases, by the hypothesis of a specific microphyte, whose ravages constitute the pathogenetic element. This tendency is exemplified in the theory, recently formulated, and ably supported by Klebs, of Prague, and Eberth, of Zurich, as to the etiology of typhoid fever.*

According to these pathologists, typhoid fever is due to a peculiar microbe which presents itself in the form of rods or filaments, some of which contain spores. Between the rods and the filaments transition forms are observed, which make it probable that the latter are derived from the former.

These micro-organisms have been found by Klebs, in accordance with the period of the disease from which death resulted, in the following localities: 1, in the intestine, first in the glands of Leiderkuhn, later in the interglandular tissue and in Peyer's patches, still later in the submucous cellular tissue, and even in the muscular layer; 2, in the mesenteric glands; 3, in the spleen; 4, in the lungs (the parts affected with hypostatic pneumonia); 5, in the brain.

He has also found them in abscesses complicating typhoid fever,

---

* *Bulletin gen. de Therapeutique*, June 30, 1883. Desplat's article, to which we are indebted for this summary.

in ulcerations of the larynx, in the kidneys, the myocardium, etc. In these various sites he has detected the same microbe, and in numerical abundance proportioned to the disorder of function of the organ where the bacillus has been found. Klebs has made many cultures of the microbe, and has inoculated animals with the final product, reproducing in hares, pigeons, guinea-pigs, etc., the characteristic anatomical lesions of typhoid fever.

Eberth, of Zurich, after an independent series of investigations, has found in the lymph-organs of the abdomen—intestinal mucous membrane, mesenteric glands, spleen—certain rods whose characters correspond with those noted by Klebs. These rods have, in fact, been identified with Klebs' bacilli.

Klebs gives a graphic *résumé* of the march of this malady. The bacillus typhosus, or its spores, are taken into the mouth and pharynx in the act of respiration, and are carried to the stomach with the saliva or food. Arriving in the small intestine, they produce by their multiplication a diffuse catarrhal inflammation, and commence to penetrate the mucous membrane. This anatomical stage corresponds to the period of incubation of the disease, a period characterized by anorexia, prostration, and a slight febrile movement. At this epoch the disease is still local; it is not till later, when the bacillus has effected an entrance into the blood and has invaded the organs, that we witness the second "classic" stage, the stage of infection, characterized by fever, cerebral symptoms, etc. At the same time the intestinal inflammatory process concentrates itself on Peyer's patches, which finally become necrosed and eliminated. The microbes, moreover, may multiply in the spleen, brain, lungs, and produce there grave disorders.

It sometimes happens that the bacillus first undergoes development in the lungs; the disease then commences as a pneumonia (typhoid pneumonia); at the autopsy the intestinal lesions (when they exist) are more recent than the pulmonary lesions.

The primitive lesions of the intestines are not always in such direct relation to the secondary lesions of other organs, that to the intestinal alterations, however extensive and profound, corresponds a proportionate perturbation of other functions of the economy. The autopsy often demonstrates that the intestinal ulcerations may be most marked and grave, at the same time that the general infection may have been almost nul; such are the cases described as *typhus ambulatorius*, or walking typhoid. On the other hand, with general infection and serious constitutional symptoms, the local intestinal lesions may be inconspicuous or wanting. In a word, typhoid fever comprehends two distinct diseases; the local intestinal lesion, and general infection with localization in different organs. These two factors of this complex disease are synchronous in a part of their duration. The first precedes the second, and the latter is in full evolution while the lesions of the former are undergoing reparation.

The germ theory of typhoid fever is far more probable than the chemical theory; in fact, we can imagine no chemical ferment or "poison" which is capable of producing such phenomena, while the hypothesis of a *contagium vivum*, infinitesimal in its commencement, pullulating in the system, and invading important vital organs, which are robbed by it of their life-sustaining oxygen and pabulum, till the environing conditions are no longer favorable for the maintenance of the parasite, well explains all the striking phenomena of the disease. Moreover, important inductive evidence is every year making the microphyte theory less an hypothesis and more a fundamental fact in pathology. The inoculation experiments of Klebs, above alluded to, constitute the kind of proof now demanded.

Almost all pathologists being convinced of the germinal origin of all contagium, there is a dominant desire on the part of therapeutists to attack the microbe by suitable germicides in the alimentary canal or in the blood, to prevent its multiplication and effect its destruction. There is very general agreement that we are not yet in possession of any sure and safe means of combating the germinal *materies morbi* when once it has effected entrance into the blood. No one has more clearly shown this than Professor Germain Sée in a late lecture.* Quantities of any known antiseptics, such as would be necessary to rid the blood of the parasitic contagium would be toxic and speedily fatal to the organism. This is notably the case with such germicides as bichloride of mercury, chlorine, iodine, carbolic, and

* Traitement de la Fièvre Typhoide, Paris, 1883.

even salicylic acid. Salicylic acid, the only acid to be seriously thought of in this connection, has never yet been given in sufficient doses to arrest typhoid fever in its march, or even materially to modify its evolution. Professor Sée has known of a rheumatic patient who, while taking ten grains every two hours of salicylic acid for several days, was nevertheless attacked by typhoid fever, which pursued its usual course.

Being then debarred from any safe and certain chemical antidotes against the morbific bacillus when it has pervaded the system, our therapeutical efforts are diverted to other indications, such as the support of the vital forces in the struggle in which they are engaged. But can we do nothing to effect the destruction of the disease-germ while it is in the alimentary canal, before it has penetrated the tissues or gained the circulating fluid? Not a few distinguished therapeutists have answered this question in the affirmative, notably Gueneau de Mussy, Herard, Hallopeau, Liebermeister, Wunderlich, and in this country, Dr. James C. Wilson and Roberts Bartholow.* The treatment of the last two authorities is essentially identical. Calomel is given in full purgative doses during the first week. This is exhibited for a double purpose, to restore heat production and to destroy typhoid germs in the intestine. Iodine is given throughout the disease, in combination with carbolic acid, or as Lugol's solution. Bartholow's formula is two parts tincture of iodine to one of strong carbolic acid; of this from one to three drops every three hours during the day and night.† By this medication it is intended to arrest the multiplication of germs in the intestine and prevent fermentation.

Professor Henri Desplats, of Lille, is favorably known by his numerous theses on the antiseptic treatment of fevers, especially by a memoir published last year on the treatment of typhoid fever by carbolic acid. After long experimentation with various salicylates in typhoid fever, he has found in the salicylate of bismuth the great desideratum. It is sparingly soluble, and therefore is

* *Vide* Wilson on Continued Fevers, p. 228. New York, William Wood & Co. Also an able statement of Wilson's and Bartholow's antiseptic method of treating typhoid fever in the *New York Medical Journal*, vol. xxxvii, page 298.

† *Boston Medical and Surgical Journal*, February 1, 1883.

more likely than most medicaments to escape absorption in the stomach and to reach the diseased intestine. It moreover has an energetic action on the organized ferments, and is a bactericide of great power. Taken during the prodromic period, when the infection is purely intestinal, the microbes not having entered the blood and tissues, its efficacy is greater; in Dr. Desplat's experience it has even had a marked abortive action. Thus, out of twenty cases reported by him, eleven (or more than one-half) treated in the first stage were cut short in four or five days under the free use of salicylate of bismuth. At least this is the belief of the professor, who gives the particulars of these cases with great minuteness, showing that the patients had all the characteristic symptoms of the disease with varying degrees of severity. In four cases the bismuth salt had a very decided moderating effect on the temperature, but the disease was prolonged by complications. In five cases, all of which were very grave, the action of the medicament was nul. The ordinary dose was about a scruple. This was repeated sufficiently often, so that the daily quantity taken should equal about six grammes (or about one drachm and a half). In some instances, where the fever-heat was excessive, the salicylate was given in one full dose of three, or even six drachms, but these large doses proved somewhat depressive. The medicament is not unpleasant to the taste and is easily borne. In short, the perusal of this article seems to justify the hope that in the salicylate of bismuth we have a new medicament of very great antiseptic value.—*Medical Record.*

